# Dimerization of the Glucan Phosphatase Laforin Requires the Participation of Cysteine 329

**DOI:** 10.1371/journal.pone.0069523

**Published:** 2013-07-26

**Authors:** Pablo Sánchez-Martín, Madushi Raththagala, Travis M. Bridges, Satrio Husodo, Matthew S. Gentry, Pascual Sanz, Carlos Romá-Mateo

**Affiliations:** 1 Instituto de Biomedicina de Valencia, CSIC and Centro de Investigación Biomédica en Red de Enfermedades Raras (CIBERER), Valencia, Spain; 2 Department of Molecular and Cellular Biochemistry, Center for Structural Biology, University of Kentucky, Lexington, Kentucky, United States of America; Stanford University, United States of America

## Abstract

Laforin, encoded by a gene that is mutated in Lafora Disease (LD, OMIM 254780), is a modular protein composed of a carbohydrate-binding module and a dual-specificity phosphatase domain. Laforin is the founding member of the glucan-phosphatase family and regulates the levels of phosphate present in glycogen. Multiple reports have described the capability of laforin to form dimers, although the function of these dimers and their relationship with LD remains unclear. Recent evidence suggests that laforin dimerization depends on redox conditions, suggesting that disulfide bonds are involved in laforin dimerization. Using site-directed mutagenesis we constructed laforin mutants in which individual cysteine residues were replaced by serine and then tested the ability of each protein to dimerize using recombinant protein as well as a mammalian cell culture assay. Laforin-Cys329Ser was the only Cys/Ser mutant unable to form dimers in both assays. We also generated a laforin truncation lacking the last three amino acids, laforin-Cys329X, and this truncation also failed to dimerize. Interestingly, laforin-Cys329Ser and laforin-Cys329X were able to bind glucans, and maintained wild type phosphatase activity against both exogenous and biologically relevant substrates. Furthermore, laforin-Cys329Ser was fully capable of participating in the ubiquitination process driven by a laforin-malin complex. These results suggest that dimerization is not required for laforin phosphatase activity, glucan binding, or for the formation of a functional laforin-malin complex. Cumulatively, these results suggest that cysteine 329 is specifically involved in the dimerization process of laforin. Therefore, the C329S mutant constitutes a valuable tool to analyze the physiological implications of laforin’s oligomerization.

## Introduction

Laforin is a 331 amino acid protein encoded by the *EPM2A* gene. Mutations in *EPM2A* result in a rare, progressive myoclonus epilepsy called Lafora disease (LD, OMIM 254780) [1], [2]. LD is an autosomal, recessive neurological disorder of devastating effects that appears at early adolescence and manifests through epileptic crises, myoclonus, tonic-clonic seizures, and a progressive dementia, apraxia, aphasia and visual loss [3]. Patients usually die within ten years of the first symptoms, and no effective clinical treatment has yet been found. At the molecular level, the most characteristic hallmark of LD is the formation of intracellular polyglucosan inclusions called Lafora bodies (LBs), first described in neurons but later found in many other tissues (mainly skeletal muscle, skin and liver) [4]. LBs are poorly branched polyglucosans that are less water-soluble than normal glycogen. Additionally, LBs contain ubiquitin and a certain amount of sequestered proteins related to the chaperone and proteasome systems [5]. Laforin has been described as a glucan phosphatase capable of removing phosphate from glycogen [6]–[9], and a direct link between laforin loss of function and LBs formation has been suggested [7], [8], [10], [11]. LBs contain excess phosphate compared to normal glycogen [7], [12]–[14]. The source of this phosphate is currently under investigation. One paper recently reported that glycogen synthase makes an error in 1/10,000 glucose units so that a phosphate moiety is introduced [15]; however, a more recent report called these results into question [16]. Nonetheless, one of the functions of laforin is to remove the phosphate so that glycogen branches appropriately and does not form LBs. However, no consensus has been reached as to whether LBs cause the neurological symptoms of LD patients, or if LBs are just the consequence of prior abnormalities affecting glycogen metabolism and protein clearance processes that drastically alter neuronal function and finally result in LBs formation [8], [17], [18].

The complexity of LD molecular etiology increased with the discovery of another affected locus, *EPM2B*, in approximately half of the cases [19], [20]. *EPM2B* encodes malin, an E3-ubiquitin ligase of the RING type. Data from multiple labs demonstrate that malin and laforin physically interact and that this interaction is required for ubiquitination of laforin itself [21] and other glycogen-related substrates such as the muscle isoform of glycogen synthase (MGS) [22], glycogen debranching enzyme (GDE) [23], and the glycogenic regulatory subunits of PP1 (type 1 protein phosphatase) both R5/PTG (Protein Targeting to Glycogen) as well as R6 [24], [25]. The implication of laforin and malin in glycogen metabolism - either by means of a direct glycogen dephosphorylation executed by laforin, by the ubiquitination of glycogenic regulators through the laforin-malin complex, or by a balanced participation of both processes - has guided research in LD over the last several years. Additionally, recent work has described novel relations between laforin, malin and other proteins involved in protein clearance systems [5], [26]–[28]. Moreover, the effects of laforin-malin abnormalities produce autophagy impairment and ER stress in animal models [29]–[32].

Laforin is classified in the family of atypical Dual Specificity Phosphatases (atypical DSPs), which belong to the larger Protein Tyrosine Phosphatase (PTP) superfamily [33], [34]. All proteins in the PTP superfamily share the presence of a signature catalytic motif that encompasses a catalytic cysteine residue (HCxxxxxRS/T, C266 in laforin). This motif is situated in a structurally conserved catalytic domain called the dual specificity phosphatase domain (DSP). The DSP domain is often accompanied by one or more accessory domains that provide different substrate specificity and interaction properties. In this sense, laforin is unique since it is the only PTP member that consists of a DSP domain attached to a carbohydrate-binding module (CBM) [35]. Recombinant laforin is an active phosphatase that can dephosphorylate phospho-tyrosine, phospho-serine/threonine and phospho-glucans *in vitro* [6], [35], [36]. However, no physiological proteinaceous substrate for laforin has yet been defined.

To date, no crystal structure of laforin has been determined and thus the specific properties and relative positions of the N-terminal CBM and the C-terminal DSP domain remain uncertain. Interestingly, laforin was found to dimerize both *in vitro* and *in vivo* and it was postulated that dimerization was a functional feature of the protein that regulated its phosphatase activity [37]. However, previous work in our lab and others showed that laforin activity does not depend on dimerization, and that laforin is present mainly as a monomer under reduced conditions [38]. We demonstrated that laforin dimerization is sensitive to redox conditions, and addition of a reducing agent to samples results in a predominance of monomeric laforin. On the contrary, oxidative conditions increase not only the formation of dimers but also of high molecular weight aggregates [39]. These redox requirements suggested that intermolecular laforin disulfide bonds may be responsible for dimerization. Thus, we decided to elucidate the specific cysteine residues that are necessary for laforin dimerization. Towards this goal, we mutated each laforin cysteine residue to a serine and tested if the Cys/Ser mutant protein could dimerize. We identified Cys329 as a residue that is necessary for laforin dimerization. Per our predictions, laforin-C329S that is unable to dimerize still maintains the main functional properties of wild type laforin: 1) the ability to bind carbohydrates, 2) phosphatase activity, and 3) the ability to bind malin and participate in malin-directed ubiquitination.

These results have accomplished two complementary goals: first, we determined that Cys329 is necessary for laforin oligomerization; and second, we have obtained an exclusively monomeric form of laforin. This monomeric form of laforin can now be utilized as a valuable tool to elucidate the specific function of laforin dimers in the pathophysiology of Lafora disease as well as being utilized in crystallographic studies to determine the tertiary structure of this protein.

## Methods

### Sequence Analysis

For the creation of a multiple amino acid sequence analysis, sequence of human laforin was used as query for the retrieval of entries from the UniprotKB database (a complete list of the sequences used is shown in Table S1). Alignment of sequences was performed using ClustalW [40] and manually edited with Bioedit [41].

### Structural Modeling of Laforin Domains

To generate a homology model of the laforin CBM and DSP, we first used BLASTp to obtain the appropriate structural templates. The sequence of each domain was analyzed via BLASTp against the PDB database. The top hit for the laforin CBM was glucoamylase (PDB ID: 1ACZ) [42] and the top hit for the DSP domain was SEX4 (PDB ID: 3NME) [43]. The sequences for the top ten hits for each domain were then aligned individually with the laforin sequence using PROMALS3D [44], and the resulting alignments were manually inspected for positional matching in residues that are known to be critical for CBM and DSP function. SWISS-MODEL was used to generate the laforin homology models, and the models were assessed using Anolea, Gromos, QMEAN6, DFire, and Verify3D [45]–[47]. Multiple models were generated and each model was analyzed to determine which BLASTp hits generated the best models. In each case, the top BLASTp hit yielded the best homology model. Images were generated using PyMol. Twenty residues in the C-terminus of the laforin DSP model, which includes Cys329, are unstructured in the model due to the absence of homology with the DSP of SEX4.

### Plasmids and Mutagenesis

Human laforin DNA was amplified using pEG202-laforin [25] as a template, using NdeI and NotI as the flanking sites; pET21b+-laforin C109S/110S, C123S, C169S, C205S, C250S, C266S, C278S and C329S were obtained by site directed mutagenesis using plasmid pET21b+-laforin as template, the Quick Change kit (Stratagene) and the corresponding mutagenic oligonucleotides. These mutagenic nucleotides contained the corresponding 21 nucleotides upstream the codon to be mutagenized and the downstream 21 nucleotides (45 nucleotides in total; see Table S2), based on the human laforin cDNA. Nucleotides in the mutated codon were replaced by the appropriated nucleotides to code for Ser. All mutants were sequenced to ensure that additional mutations were not introduced during the mutagenesis procedure. Similarly, plasmid pCMV-HA laforin (C329S) was obtained by site directed mutagenesis using plasmid pCMV-HA laforin [48] as template, the Quick Change kit (Stratagene) and the corresponding mutagenic oligonucleotides aforementioned. Plasmids pCDNA3-HA-malin and pCMV-myc-R5/PTG are described in [25] and pCMV-6xHisUbiq was from Dr. Manuel Rodriguez, Proteomics Unit, CIC-BioGUNE, Vizcaya, Spain. Plasmids pACT2-malin and pEG202-laforin are described in [25].

### Protein Purification and Phosphatase Assays

*Escherichia coli* DH5α was used as the host strain for plasmid constructions. *E. coli* BL21 (RIL) was used for protein production. They were grown in LB (1% peptone, 0.5% yeast extract, 1% NaCl, pH 7.5) medium supplemented with 50 g/L ampicillin and 30 g/L chloramphenicol. Transformants were grown at 37°C until the absorbance at 600 nm reached a value of around 0.3. IPTG was then added to a final concentration of 0.1 mM, and cultures were maintained at 37°C for 3 h. Cells were harvested and resuspended in 20 mL of sonication buffer [100 mM Tris-HCl pH 7.5, 150 mM NaCl, 2 mM PMSF and complete protease inhibitor cocktail (Roche)]. Cells were disrupted by sonication and the fusion proteins purified by passing the extracts through columns containing 1 mL bed volume of amylose resin (N.E. Biolabs). Recombinant proteins were eluted from the column with 500 mM maltose. Gel filtration chromatography was performed using an AKTA Purifier with a HiLoad 16/60 Superdex 200 10/300 GL size exclusion column (GE Healthcare).

*In vitro* phosphatase assays were performed using 4 µg of recombinant proteins diluted in phosphatase buffer (0.1 M Tris-HCl, 40 mM NaCl, 10 mM DTT), in the presence of 0.5 mM 3-O-methyl fluorescein phosphate (OMFP, Sigma). Reactions were carried out at 37°C in a final volume of 200 µl in 96-well ELISA plates. Phosphatase activity was measured for 3 hours as absorbance at 490 nm. One Unit of phosphatase activity is defined as the one unit of change in the absorbance at 490 nm per min of assay. Values are expressed as specific activity (Units/mg of protein).

### Yeast Two-hybrid Analysis

Yeast THY-AP4 strain [MAT*α ura3 leu2 trp1 lexA::lacZ lexA::HIS3lexA::ADE2*] [49] was transformed with plasmids pACT2-malin (GAD-malin) and pEG202-laforin (LexA-laforin) or pEG202-laforin-C329S (LexA-laforin-C329S). Transformants were grown in selective SC medium, and β-galactosidase activity was assayed in permeabilized cells and expressed in Miller units as in [50].

### Cell Culture and Immunodetection

Human embryonic kidney (HEK293) cells were grown in DMEM (Lonza) supplemented with 100 units/mL penicillin, 100 µg/mL streptomycin, 2 mM glutamine, 10% inactivated fetal bovine serum (GIBCO). 1.5×10^6^ cells were plated onto 60 mm culture dishes the day before transfection. Cells were transfected with 1 µg of pCMVmyc-laforin plasmid using Lipofectamine 2000 (Invitrogen). Twenty-four hours after transfection, cells were scraped on ice in lysis buffer [10 mM Tris-HCl pH 8; 150 mM NaCl, 15 mM EDTA; 0.6 M sucrose, 0.5% nonidet P-40 (NP-40), protease inhibitor cocktail (Roche), 1 mM PMSF, 50 mM NaF and 5 mM Na_2_P_2_O_7_]. Cells were lysed by repeated passage through a 25-gauge needle. To analyze proteins under non-denaturing, non-reducing conditions, cell extracts (25 µg) were diluted in SDS- and DTT-free loading buffer (125 mM Tris-HCl, 20% glycerol) [38] and analyzed by regular SDS-PAGE and immunoblotting using an anti-myc, anti-HA (Sigma) or anti-laforin [25] antibody.

### Co-immunoprecipitation Analysis

HEK293 cells were transfected with pCMV-HA-malin and pCMV-myc-laforin or pCMV-myc-laforin-C329S, or with the corresponding empty plasmid. Cells were scraped on ice in lysis buffer [50 mM Tris-HCl pH 8; 10 mM KCl, 50 mM EDTA; 15% glycerol, 1% nonidet P-40 (NP-40), complete protease inhibitor cocktail (Roche Diagnostics, Barcelona, Spain), 1 mM PMSF, 50 mM NaF, 2 mM NaVO4 and 5 mM Na_2_P_2_O_7_]. Cell lysates were then centrifuged at 13,000×g for 15 min at 4°C. Laforin-malin complexes were immunoprecipitated from supernatants (800 µg of total protein) with anti-myc polyclonal antibody (Sigma–Aldrich, Madrid, Spain) and visualized by immunoblotting using anti-myc or anti-HA (Sigma–Aldrich, Madrid, Spain) antibodies.

### Analysis of Ubiquitination

To study ubiquitination in intact cells, HEK293 cells were transfected with plasmids pCMV-6xHisUbiq (encoding a modified ubiquitin, tagged with 6xHis residues), plasmids encoding laforin and the E3-ubiquitin ligase malin and pCMVmyc plasmids encoding the protein of interest, using the Lipofectamine 2000 reagent (Invitrogen, Madrid, Spain), according to the manufactureŕs instructions. After 36 hours of transfection, cells were lysed in buffer A (6 M guanidinium-HCl, 0.1 M sodium phosphate, 0.1 M Tris-HCl, pH 8.0). 1 mg of protein of a clarified extract (CE; 12,000×g, 15 min) were incubated in 100 µl TALON column (Clontech, Barcelona, Spain) in the presence of 10 mM imidazole, for 3 hours at room temperature on a rocking platform, to purify His-tagged proteins. The column was then successively washed with 2 mL each of buffer B (buffer A plus 10 mM imidazole), buffer C (buffer B, but with 8 M urea instead of 6 M guanidinium-HCl) and four more times with buffer C adjusted to pH 6.0. Bound proteins were eluted with 50 µl of 2× Laemmli’s sample buffer and analyzed by Western blotting using appropriate antibodies.

### Statistical Analysis

Values are given as means ± SD of at least three independent experiments. Differences between phosphatase activities of wt laforin and mutant forms are analyzed by two-tailed student’s t-test. The significance has been considered at *p<0.05, **p<0.01 and ***p<0.001, as indicated in each case. Data for protein purification, gel electrophoresis, and ubiquitination is representative of at least three independent determinations.

## Results

### Identification and Mutagenesis of Cysteine Residues

**Figure 1:**
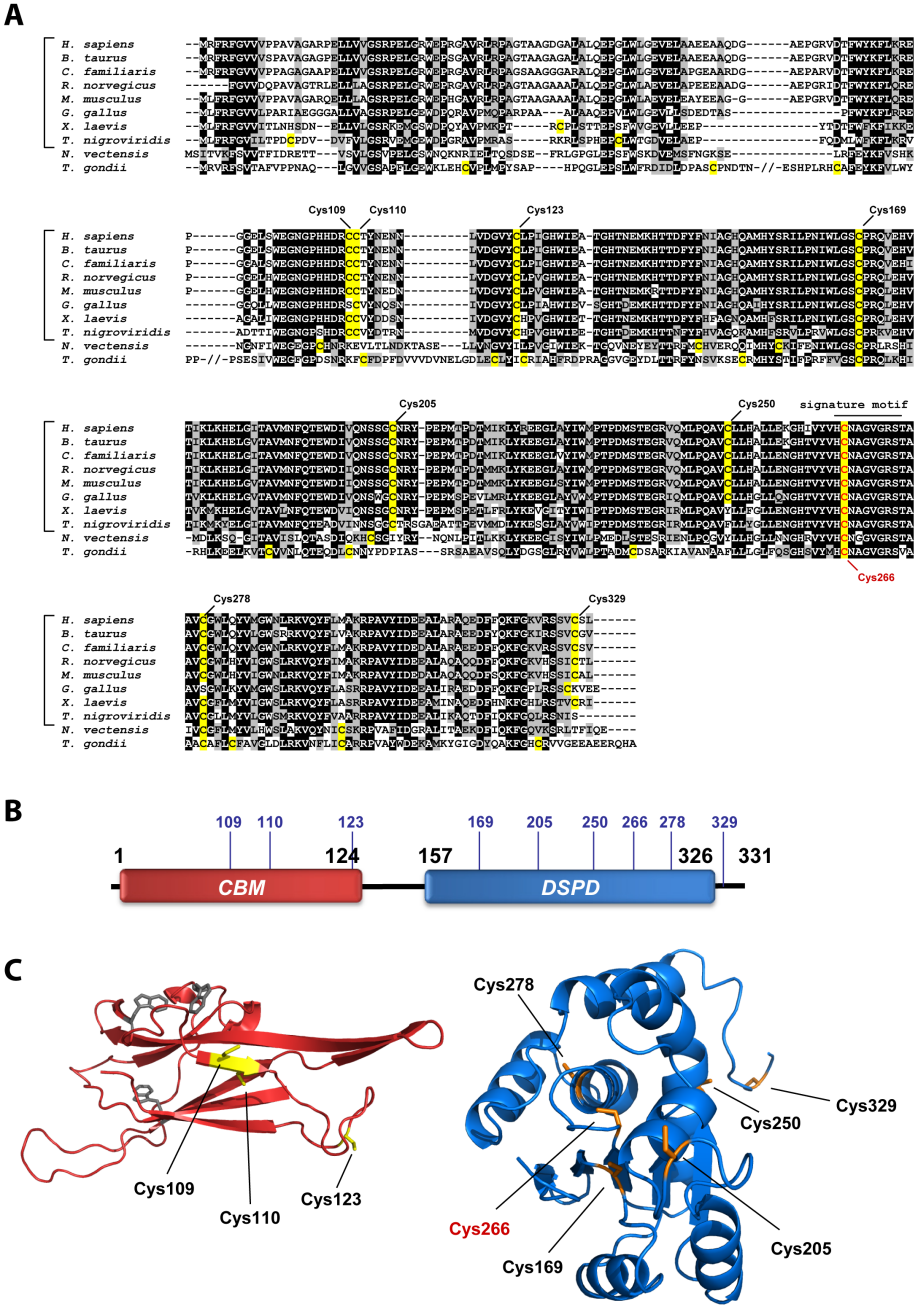
Human laforin contains nine cysteines. (A) Multiple sequence alignment of laforin orthologs. Sequences of several vertebrate (indicated by the brackets) and two invertebrate (*N. vectensis* and *T. gondii*) organisms were used (see Table S1 for details). The different cysteines are highlighted in yellow, and the position of cysteines in human laforin is marked (catalytic cysteine C266 appears in red). (B) Schematic of laforin domains (CBM: carbohydrate binding module; DSPD: dual-specificity phosphatase domain). The location of the nine cysteines is shown (blue numbers). (C) Tertiary structure prediction of the CBM (left) and DSPD (right) laforin domains. Homology models were created using the structures of glucoamylase (PDB: 1ACZ) and SEX4 (PDB: 3NME) as templates for the CBM and DSPD, respectively. The models were used to estimate the possible location of the cysteines in the tertiary structure. The cysteines studied in this work appear in yellow in the CBM and in orange in the DSPD; in grey, known tryptophans responsible of the carbohydrate binding.

We first sought to define the relative position and degree of evolutionary conservation for each cysteine residue in human laforin. Therefore, we generated a multiple sequence alignment of laforin orthologs from different phyla. Laforin has an interesting evolutionary lineage that has been previously thoroughly described [9], [51]. A gene encoding laforin is conserved in all analyzed vertebrate genomes, but the laforin gene is absent from most invertebrate and protozoan genomes, including the common model organisms of yeast, worm, and fly. However, a gene encoding laforin is found in the cnidarian *Nematostella vectensis* and the protozoan *Toxoplasma gondii*. Based on the previously described phylogeny, amino acid sequences of laforin orthologs from several vertebrate organisms, one cnidarian, and one protozoon (see Methods for a list of entries) were selected and aligned using ClustalW. The resulting alignment shows a high degree of conservation, especially in the DSP domain. Human laforin contains nine cysteine residues, three of them located in the CBM (C109, C110 and C123) and six in the DSP (C169, C205, C250, C266, C278 and C329) (**Figure 1**). Only the position of C169 and C266 is conserved in all sequences, although most of the vertebrate orthologs share all nine cysteines along their primary sequence (**Figure 1A**). C266 is the critical catalytic activity residue [35], and it has previously been described that mutation of it to Serine (C266S) abolishes phosphatase activity, but does not affect laforin dimerization [38], [52].

Next, we generated homology models (see Methods) to estimate the location and solvent accessibility of each cysteine residue. The cysteine residues located in the CBM appear located in a surface exposed site and far from the residues involved in carbohydrate binding (**Figure 1C**). In the DSP domain, C169, C205, C250 and C329 are predicted to be located in a more surface exposed location, whereas the rest of residues are predicted to be buried in the structure. Since neither the primary sequence nor the tertiary structure analysis suggested a clear and unique candidate residue responsible for dimerization, and in order to determine the implication of each cysteine in the formation of dimers, we created laforin mutants that changed individual cysteine residues to serine. With this strategy we obtained eight mutant forms: seven individual mutations (C123S, C169S, C205S, C250S, C266S, C278S and C329S) and one double mutation (C109S/110S). This latter form was created in order to avoid a possible masking effect of cysteines C109 and C110 because of their close proximity. The mutant proteins were then expressed in *E. coli* with no epitope tag and recombinant mutant proteins were purified by affinity purification using an amylose resin. This methodology presents two advantages: first, it avoids the necessity of adding any tag in the purified protein, due to the natural capacity of laforin to bind carbohydrates; and second, it serves as a test of CBM functionality in each laforin mutant. All mutant proteins were successfully purified using this strategy with the exception of C278S. In this case, we were unable to obtain any recombinant protein from *E. coli*. In fact, laforin-C278S did not even express in *E. coli* and we did not observe laforin-C278S in either the soluble or the pellet fraction from the bacterial extracts. We also attempted to express laforin-C278S in a yeast model but again, we were unable to recover laforin-C278S from this system either. For this reason, the mutant form of laforin C278S was not further studied in this work. The rest of the seven mutants resulted in soluble proteins that all bound the amylose resin.

### Functional Analysis of Cysteine Mutants

**Figure 2:**
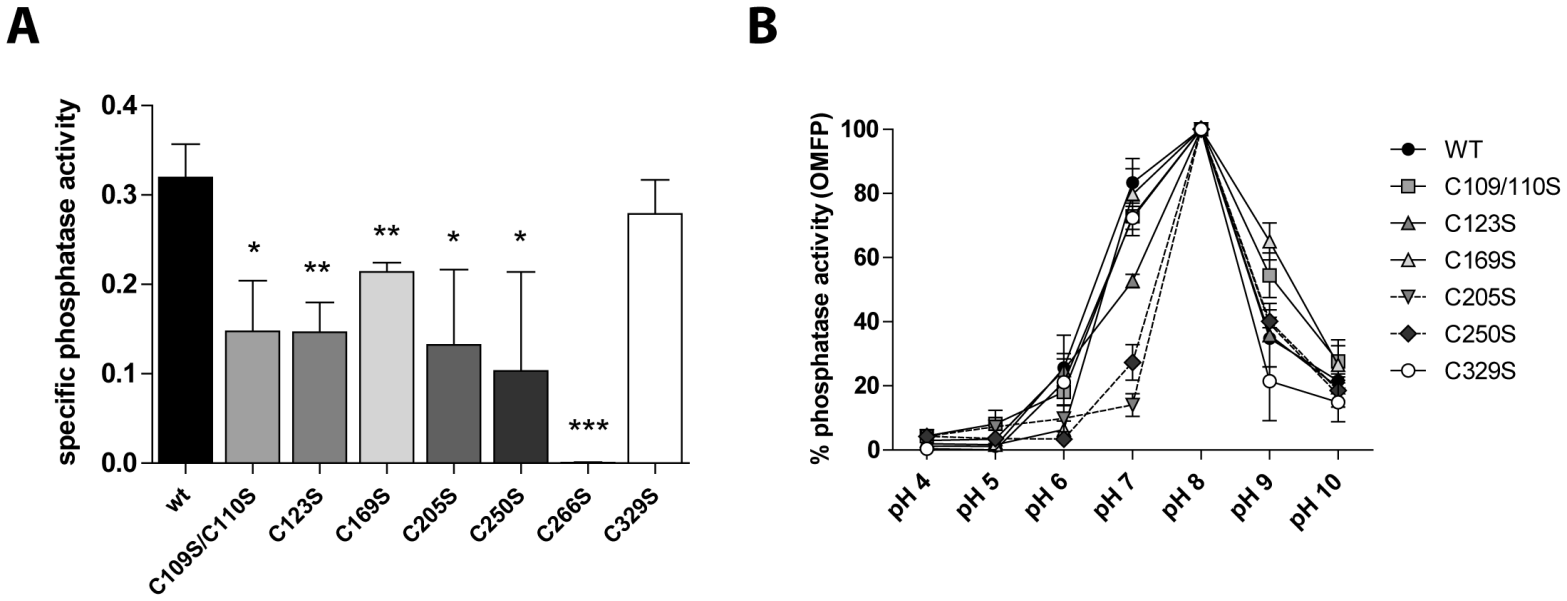
Functional analysis of laforin mutants. (A) Recombinant proteins expressed in bacteria were purified using an amylose resin and used for *in vitro* phosphatase assays employing the artificial substrate OMFP. The mutant C266S was used as a negative control. (B) The same samples were used in phosphatase assays using different pH conditions and represented as % phosphatase activity compared to the pH of maximum activity of each mutant (pH = 8 in all cases). Error bars represent SD of three independent measurements; statistical significance refers to the activity of wt protein (*p<0.05, **p<0.01 and ***p<0.001).

Since each of the seven laforin mutants that were expressed in *E. coli* all bound the amylose resin, we concluded that laforin cysteines in either the CBM or the DSP domain are not necessary to promote carbohydrate binding. This result is not surprising since glucan binding is often driven by aromatic residues in CBMs [35], [53]. Next, we wanted to determine the integrity of the DSP domain of each mutant using *in vitro* phosphatase assays with the artificial substrate OMFP. Wild type and C266S mutant were used as positive and negative controls of activity, respectively. As shown in **Figure 2A** **,** most of the laforin mutants displayed decreased phosphatase activity with the exception of C329S that exhibited a similar activity to wild type. All mutants dephosphorylated OMFP with a maximum catalytic efficiency at pH 8, although mutant forms C205S and C250S showed a stronger sensitivity to pH changes (**Figure 2B**).

### Recombinant Laforin-C329S Mutant is Present as a Monomer

**Figure 3:**
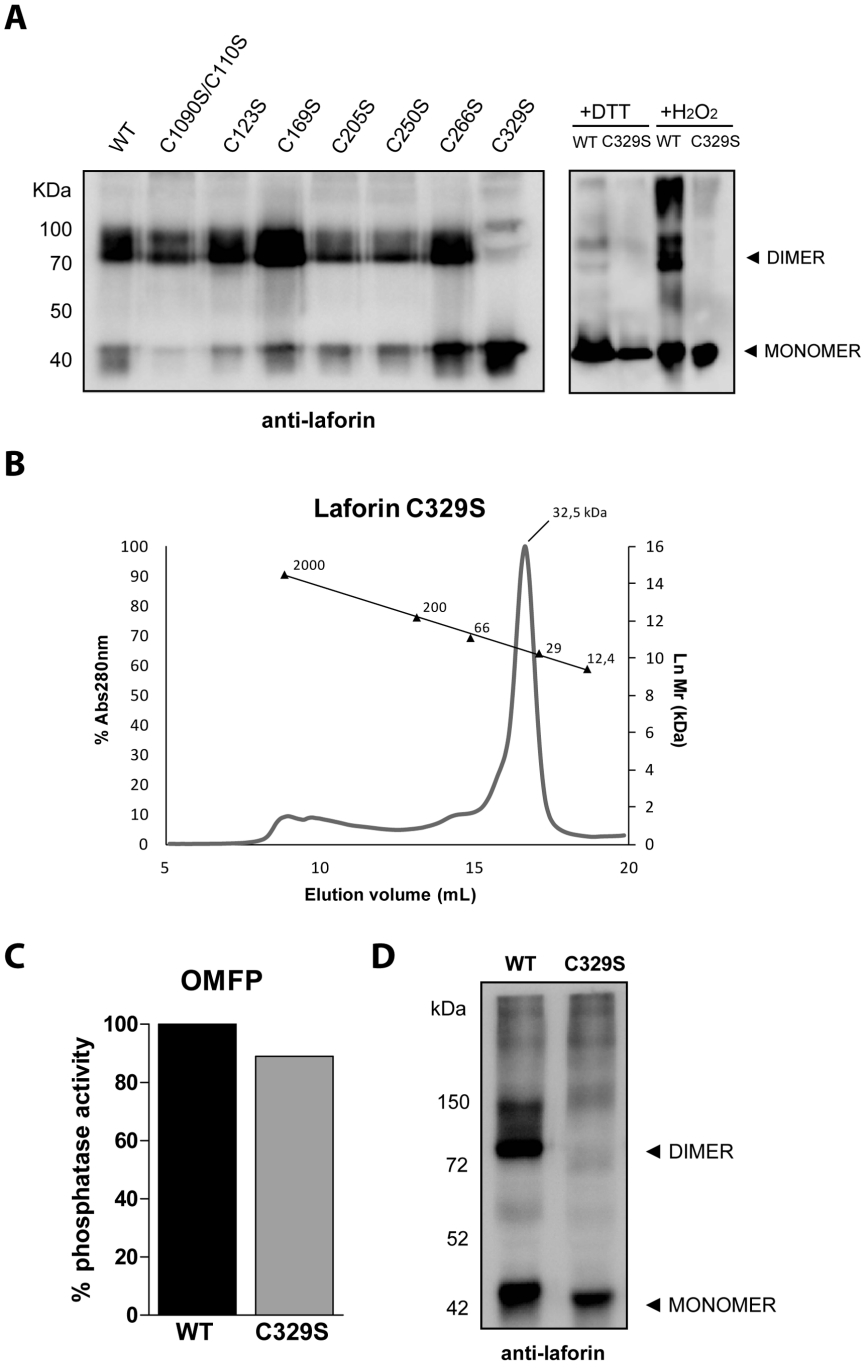
Laforin-C329S is monomeric. (A) I*n vitro* dimerization assay. Recombinant proteins expressed in bacteria were purified and subjected to non-reducing, non-denaturing electrophoresis and were immunodetected using anti-laforin antibody. All mutants could form dimers with the exception of C329S (left panel). WT laforin and C329S were further analyzed by incubation in the presence of 10 mM DTT or 10 mM H_2_O_2_ and analyzed for the presence of monomeric and dimeric species (right panel). (B) Laforin C329S exclusion chromatography analysis. A single peak is observed in an elution volume corresponding to 32.5 kDa (molecular size markers are indicated). (C) *In vitro* phosphatase activity assay of the same sample showed that the C329S mutant displayed a catalytic activity comparable to the wild type protein. (D) *In vitro* dimerization assay of laforin-C329S expressed in mammalian cells. Crude lysates from HEK293 cells transfected with myc-laforin (wt, wild type) or myc-C329S plasmids were analyzed by Western blot. The electrophoresis was carried out in non-reducing, non-denaturing conditions, and the immunodetection was performed using anti-laforin antibody. The position of the monomeric and dimeric forms of laforin is indicated.

After defining the ability of the mutants to bind glucans and determining their phosphatase activity, we defined the dimerization capacity of each mutant. Previous studies have reported that dimerization of recombinant laforin can be assayed by electrophoresis in non-denaturing, non-reducing conditions (see Methods). Purified recombinant protein samples were analyzed by this method, Western blotted, and detected using anti-laforin antibody (**Figure 3A**). Interestingly, all mutant forms were able to dimerize, with the exception of C329S that was present exclusively as a monomer. Therefore, we further compared the oligomerization capacity of wild type laforin and the C329S mutant form. Laforin WT and C329S were first incubated with either a reducing agent (DTT) or an oxidizing agent (hydrogen peroxide) and then analyzed in the same way as described above. As expected, wild type laforin appeared as an exclusively monomeric species in the presence of DTT, but as a multimeric conglomerate of species in the presence of hydrogen peroxide (**Figure 3A**, right panel). Conversely, laforin-C329S appeared as an exclusively monomeric form in both conditions (**Figure 3A**, right panel). To confirm the absence of dimerization for laforin-C329S, we performed size-exclusion chromatography. Laforin-C329S produced in bacteria was purified using amylose-resin as described above. Immediately after purification, the sample was loaded onto an analytical size-exclusion column (Superdex 200) using a FPLC. Laforin-C329S eluted as a single peak corresponding to a relative molecular weight of 32.5 kDa, coincident with the estimate for monomeric laforin (**Figure 3B**). A sample of the fraction corresponding to this peak was then used in a non-reducing PAGE where we confirmed that no dimeric forms appeared. After purifying monomeric laforin-WT and laforin-C329S we then tested the phosphatase activity of each protein and found that laforin-C329S had phosphatase activity comparable to monomeric laforin-WT (**Figure 3C**).

Next, we sought to determine if laforin-C329S could dimerize in a cell culture model. Laforin-C329S was overexpressed in HEK293 mammalian cells, cells were lysed, and crude extracts were subjected to non-denaturing, non-reducing electrophoresis as described above. As shown in **Figure 3D**, wild type protein clearly forms both a monomeric and dimeric species, whereas laforin-C329S only appears as a monomer. These results closely mimic the data observed from bacterial expression. Cumulatively, these data demonstrate that laforin-C329S displays reduced dimerization while maintaining carbohydrate binding and phosphatase activity.

### Recombinant Laforin-C329X does not Dimerize but Maintains Carbohydrate Binding and Phosphatase Activity

To further explore the effect of mutating Cys329 of laforin, we generated a laforin construct lacking the last three amino acids, laforin-C329X. Unlike the other constructs, laforin-C329X was expressed with an amino-terminal HIS_6_ epitope tag that was used during Ni-NTA purification.

**Figure 4:**
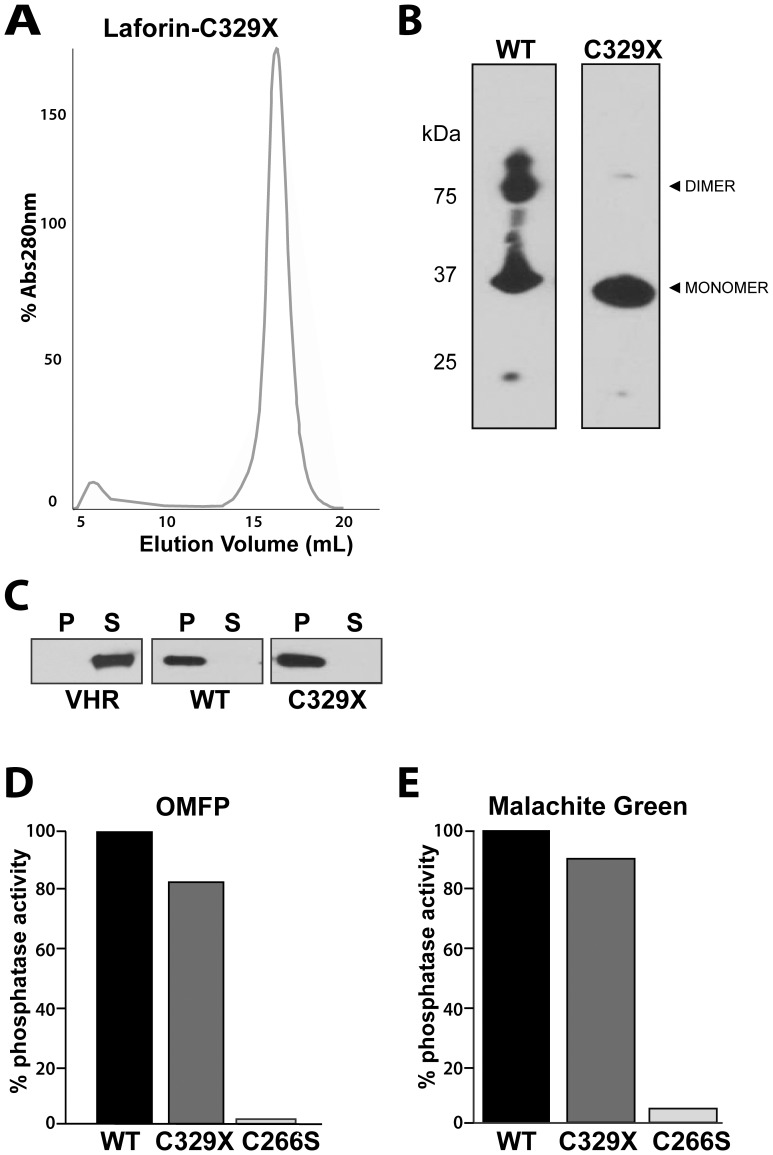
Laforin-C329X does not dimerize, but maintains activity. (A) Laforin-C329X exclusion chromatography chromatogram. Recombinant laforin-C329X was purified via affinity chromatography and then subjected a HiLoad 16/60 Superdex 200 sizing column. A single peak elutes that corresponds to 32.5 kDA. (B) *In vitro* dimerization assay. Recombinant laforin-WT and laforin-C329X were purified via affinity chromatography, subjected to non-reducing, non-denaturing electrophoresis, and immunoblotted using anti-HIS antibody. (C) Co-sedimentation assay of protein and amylopectin (amylopectin binding assay). Recombinant histidine-tagged proteins were incubated with 5 mg/ml amylopectin, amylopectin was pelleted by ultracentrifugation, proteins in the pellet (P) and supernatant (S) were separated by SDS-PAGE, and visualized by Western analysis. Amylopectin-bound proteins are found in the pellet (P) and unbound proteins are found in the supernatant (S).VHR, *Vaccinia* virus phosphatase VH1-related; WT, laforin; C329X, laforin-C329X. (D) Recombinant laforin-WT, laforin-C329X, and laforin-C266S were purified and used for *in vitro* phosphatase assays employing the artificial substrate OMFP. The mutant C266S was used as a negative control. (E) Recombinant laforin-WT, laforin-C329X, and laforin-C266S were purified and used for *in vitro* phosphatase assays employing the phosphorylated glucan amylopectin. Inorganic phosphate release was detected via malachite green.

Similar to laforin-C329S, laforin-C329X eluted from the size exclusion Superdex 200 column as a single, monomeric peak of ∼32.5 kDa (**Figure 4A**). Alternatively, wild type laforin with an amino-terminal HIS_6_ epitope eluted as two peaks. We then analyzed wild type and laforin-C329X under non-reducing PAGE conditions and found that wild type laforin formed a monomer and dimer, while laforin-C329X was largely monomeric (**Figure 4B**).

After determining that laforin-C329X behaved similar to laforin-C329S with respect to dimerization, we then sought to determine the biochemical properties of laforin-C329X. First, we tested if laforin-C329X could bind glucans. We utilized a previously established glucan-binding assay for these experiments [9], [51], [54]. As a negative control, we utilized the human phosphatase *Vaccinia* virus phosphatase VH1-related (VHR), a prototypical protein phosphatase that does not bind amylopectin [9], [54], [55]. Recombinant proteins were incubated with amylopectin, the amylopectin was then pelleted by ultracentrifugation, and proteins in the pellet (P) and supernatant (S) were visualized by Western analysis. As expected, VHR was found in the supernatant whereas wild type laforin, which possesses robust glucan binding, is in the pellet with the amylopectin (**Figure 4C**). Laforin-C329X also robustly binds amylopectin, and similar to wild type laforin is in the pellet. Next, we tested the phosphatase activity of laforin-C329X and found that it too possesses similar phosphatase activity against OMFP as wild type laforin (**Figure 4D**). Lastly, we tested the ability of laforin-C329X to remove inorganic phosphate from the phosphorylated glucan amylopectin. Potato amylopectin contains levels of mono-esterified phosphate that when released as inorganic phosphate can be detected using malachite green assay. This method measures the biologically relevant activity of laforin, i.e. its glucan phosphatase activity [6], [9], [55]. Per our predictions, laforin-C329X dephosphorylated amylopectin to a similar extent as wild type laforin (**Figure 4E**). Therefore, laforin-C329X exhibits a dramatic decrease in dimerization compared to wild type laforin while maintaining wild type levels of glucan binding and both generic and biologically relevant phosphatase activities.

These results confirm that mutation of Cys329 abrogates the propensity of laforin to form dimers. This finding prompted us to assess the effects of mutating Cys329 using a mammalian cell model in order to define the implications of laforin oligomerization in a more biologically relevant system.

### Laforin C329S Forms a Functional Complex with Malin in Mammalian Cells

As discussed in the introduction, laforin forms a complex with the E3 ubiquitin ligase malin that ubiquitinates multiple substrates involved in glycogen metabolism. We previously defined that laforin dimerization was not required for the interaction between laforin and malin [38]. Therefore, we reasoned that co-expression of laforin C329S and malin should result in normal ubiquitination of the substrates defined for the laforin-malin complex, such as protein targeting to glycogen R5/PTG [25].

**Figure 5:**
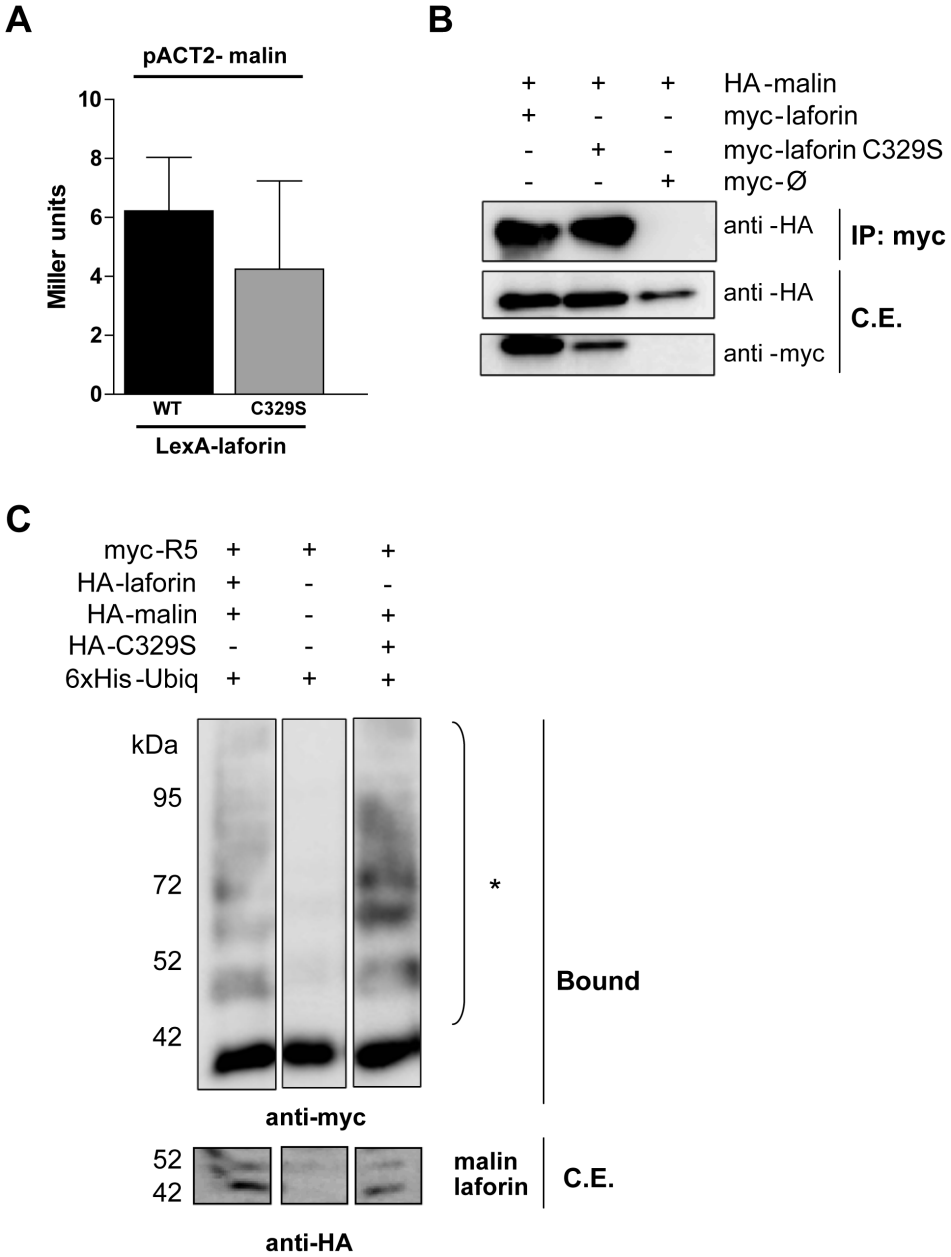
Laforin-C329S and malin physically interact and form a functional complex in mammalian cells. (A) Yeast two-hybrid analysis. THY-AP4 yeast strain was transformed with pACT2-malin and LexA-laforin (WT or C329S) and the interaction was assessed by measuring the β-galactosidase activity. (B) Co-immunoprecipitation assay. HEK293 cells were co-transfected with plasmids myc-laforin (WT or C329S) and HA-malin. Cells were lysed and total lysates were incubated with anti-myc antibody and protein A/G beads. After washing, beads were boiled in loading buffer and purified proteins analyzed by SDS-PAGE and Western blot using anti-myc or anti-HA antibodies. (C) Ubiquitination analysis of R5/PTG by the laforin-malin complex. Overexpression of 6xHis-ubiquitin, pCMV-HA-malin, pCMV-myc-R5/PTG and pCMV-HA-laforin (wild type or C329S) in HEK293 cells, followed by lysis in presence of guanidinium chloride and purification of the ubiquitinated proteins by affinity chromatography using a cobalt resin. The result of the purification was analyzed using Western blot with anti-myc antibodies. Bound: proteins retained in the resin; crude extracts (50 µgr, C.E.) were immunodetected with anti-HA antibodies. *: polyubiquitinated forms.

First, we proceeded to test if laforin-C329S was still able to physically interact with malin. We analyzed this interaction by yeast two-hybrid analysis and found no significant differences between the interaction of laforin WT and C329S with malin (**Figure 5A**). Next, we co-transfected HEK293 cells with HA-malin and myc-laforin (WT or C329S). Both laforin forms were able to co-immunoprecipitate HA-malin, corroborating that mutation of Cys329 does not affect the physical interaction between laforin and malin (**Figure 5B**). Then, we transfected HA-tagged laforin WT or C329S into HEK293 cells together with HA-malin, myc-tagged R5/PTG [25] and a HIS_6_-tagged ubiquitin plasmid. Ubiquitinated proteins from crude extracts were purified using a metal affinity resin as previously described [56] and the ubiquitination of the substrate was analyzed by Western analysis. Ubiquitination occurred in a malin-dependent manner when either wild type laforin or laforin-C329S was present; however, no R5/PTG ubiquitination occurred in the absence of both wild type laforin and laforin-C329S (**Figure 5C**). This result demonstrates that laforin-C329S, that does not dimerize, is still able to form a functional complex with malin and promotes the ubiquitination of R5/PTG.

## Discussion

Laforin, the dual-specificity phosphatase mutated in Lafora disease, is intimately involved in mechanisms leading to abnormal glycogen accumulation, autophagy impairment, and other molecular abnormalities that underlie Lafora disease. However, many biochemical and functional properties of laforin remain largely unknown. Dual-specificity phosphatases are a broad family of proteins that share little sequence conservation at the primary amino acid level, but share a highly conserved catalytic domain with respect to structure. Laforin is a unique DSP since it is the only human protein of the group that contains a carbohydrate-binding module. These unique attributes allow laforin to bind and dephosphorylate glycogen. Additionally, laforin functions in a complex with malin, and this complex participates in other cellular functions besides dephosphorylation of glycogen.

The lack of structural data for laforin has constituted a severe difficulty in assessing many of its particular features. For this reason, indirect data regarding the structure and biochemical properties of the domains that constitute the modular structure of laforin are extremely useful. Like other structurally related small phosphatases [57], laforin was reported to form dimers. However, we recently described that laforin dimerization depends on redox conditions, suggesting the involvement of disulfide bridges and hence the participation of cysteine residues in the dimerization of laforin [38]. In the present work, we have demonstrated that cysteine 329 in human laforin is necessary for the formation of laforin dimers, both in bacterial and mammalian cell models, and that dimerization of laforin is not required for its binding to carbohydrates, its phosphatase activity, or ubiquitination of R5/PTG by the laforin-malin complex.

Laforin dimerization is also observed in a functional equivalent of laforin from plants called SEX4 [38]. Interestingly, SEX4 also contains a Cys residue near its carboxy-terminus [43]. The conservation of this mechanism across kingdoms as well as the abundance of cysteines and the high conservation of the residues flanking the cysteines suggested structural roles in which the thiol group of the cysteine could be involved in intra- or inter-molecular interactions. To assess if any of these residues participate in the dimerization process, we performed site-directed mutagenesis of each cysteine and changed each one to a serine. The Cys/Ser mutation avoids the presence of a thiol group while maintaining structural and chemical characteristics of the residue. C329S was the only mutant that demonstrated impaired dimerization. Cys329 was observed in the majority of laforin orthologs examined and its position near the carboxy-terminus of the protein makes it a good candidate to form intermolecular disulphide bridges.

Recombinant laforin-C329S subjected to affinity purification and size-exclusion chromatography resulted in an exclusively monomeric form. Mutation of laforin Cys329 to Ser does not disrupt overall protein structure because we demonstrate that laforin-C329S maintains near wild type levels of phosphatase activity and it maintains its ability to bind an amylose resin. In addition to the experiments performed with laforin-C329S, we also generated a truncated form of the protein. Laforin-C329X was also a monomeric protein after size-exclusion chromatography that retained the ability to bind glucans and the catalytical properties of the wild type laforin against both OMFP as well as against the phosphorylated glucan amylopectin. This suggests that in humans, laforin C329 is necessary for the formation of dimers. C329 is highly conserved among vertebrate orthologs, but it is not strictly conserved outside of vertebrates. Thus, it may be possible that laforin dimerization represents a functional aspect of laforin in vertebrates. However, the functional equivalent of laforin in plants, SEX4, has also been shown to dimerize [38]. Thus, it is possible that dimerization is a conserved mechanism of glucan phosphatases.

Laforin functionality is defined by two biochemical properties: glucan phosphatase activity and the capacity to bind carbohydrates. Mutation of eight cysteine residues to serine did not impair the ability of the mutant proteins to bind the amylose resin. Therefore, these eight mutations did not affect the overall structure of laforin. Mutation in the additional cysteine residue C278 produced forms highly unstable that precluded its further analysis, likely due to the predicted location of C278 deep within one of the core helices structurally critical for the catalytic domain. When the phosphatase activity of the eight mutants was analyzed, a significant decrease in the catalytic activity of all mutant forms, with the exception of C329S, was found, suggesting that abrogation of thiol groups may affect the adequate conditions for the full catalytic performance of laforin. The strict pH requirement of catalytically impaired mutants, such as C205S and C250S, further corroborates this observation.

Cumulatively, the data demonstrate that laforin cysteine 329 abrogates the capacity of laforin to form dimers without affecting its ability to bind carbohydrates or its phosphatase activity. Oligomerization of proteins may constitute a critical event to define their interactions with other binding partners. Since the physiological function of laforin in regulating glycogen synthesis is tightly linked to its interaction with the E3-ubiquitin ligase malin, we sought to determine if abrogation of laforin dimerization would impede the interaction between malin and laforin. We found that laforin-C329S binds to malin with the same efficiency as the wild type protein, both in yeast and mammalian cell models. Moreover, we found that laforin-C329S was able to ubiquitinate R5/PTG in the same way as wild type protein, indicating that no dimerization is required for the formation of a functional laforin-malin complex.

The implications of this finding are of importance, since no data about the physiological implications of laforin oligomerization have previously been defined. Laforin-C329S constitutes a valuable tool to assess which laforin properties are dependent on dimerization. Our data demonstrate that both wild type and laforin-C329S maintain intact catalytic activity, ability to bind carbohydrates, and the ability to interact with malin. In addition to defining some of the physiological attributes of monomeric laforin, the laforin-C329S and laforin-C329X may prove useful for structural analysis. To date, the only structure of a glucan phosphatase is of the plant protein SEX4 [43]. The exclusion chromatography data of laforin-C329S and laforin-C329X demonstrate that they are easily purified as monomeric species. Historically, laforin has been very difficult to purify and crystallize in large part due to its propensity to oligomerize and thus produce heterogeneity in crystallographic screens. These issues have delayed the success in determining the structure of the protein. Laforin-C329S and –C329X, which are homogeneously monomeric, may aid in obtaining laforin crystals and provide a most valuable advance in our understanding of the molecular basis of Lafora disease.

## Supporting Information

Table S1 **List of entries used for sequence analysis.** Left column shows the UniprotKB ID label for the laforin orthologs used in the sequence alignment. Right column shows the species name corresponding to each protein ID.
(DOCX)

Table S2 **Primers used for mutagenesis analysis.** Mutagenesis was performed by PCR reactions combining laforin terminal primers (upper panel) with internal primers harboring each mutation (down panel). Sequences are written in 5′–3′ sense.
(DOCX)

## References

[1] Minassian BA, Lee JR, Herbrick JA, Huizenga J, Soder S, et al (1998) Mutations in a gene encoding a novel protein tyrosine phosphatase cause progressive myoclonus epilepsy. Nat Genet 20: 171–174. 9771710 10.1038/2470

[2] Serratosa JM, Gomez-Garre P, Gallardo ME, Anta B, de Bernabe DB, et al (1999) A novel protein tyrosine phosphatase gene is mutated in progressive myoclonus epilepsy of the Lafora type (EPM2). Hum Mol Genet 8: 345–352. 9931343 10.1093/hmg/8.2.345

[3] Monaghan TS, Delanty N (2010) Lafora disease: epidemiology, pathophysiology and management. CNS Drugs 24: 549–561. 20527995 10.2165/11319250-000000000-00000

[4] Minassian BA (2001) Lafora’s disease: towards a clinical, pathologic, and molecular synthesis. Pediatr Neurol 25: 21–29. 11483392 10.1016/s0887-8994(00)00276-9

[5] Rao SN, Maity R, Sharma J, Dey P, Shankar SK, et al (2010) Sequestration of chaperones and proteasome into Lafora bodies and proteasomal dysfunction induced by Lafora disease-associated mutations of malin. Hum Mol Genet 19: 4726–4734. 20858601 10.1093/hmg/ddq407

[6] Worby CA, Gentry MS, Dixon JE (2006) Laforin, a dual specificity phosphatase that dephosphorylates complex carbohydrates. J Biol Chem 281: 30412–30418. 16901901 10.1074/jbc.M606117200 PMC2774450

[7] Tagliabracci VS, Turnbull J, Wang W, Girard JM, Zhao X, et al (2007) Laforin is a glycogen phosphatase, deficiency of which leads to elevated phosphorylation of glycogen in vivo. Proc Natl Acad Sci U S A 104: 19262–19266. 18040046 10.1073/pnas.0707952104 PMC2148278

[8] Gentry MS, Dixon JE, Worby CA (2009) Lafora disease: insights into neurodegeneration from plant metabolism. Trends Biochem Sci. 10.1016/j.tibs.2009.08.002 PMC2805077 19818631

[9] Gentry MS, Dowen RH 3rd, Worby CA, Mattoo S, Ecker JR, et al (2007) The phosphatase laforin crosses evolutionary boundaries and links carbohydrate metabolism to neuronal disease. J Cell Biol 178: 477–488. 17646401 10.1083/jcb.200704094 PMC2064834

[10] Wang W, Parker GE, Skurat AV, Raben N, DePaoli-Roach AA, et al (2006) Relationship between glycogen accumulation and the laforin dual specificity phosphatase. Biochem Biophys Res Commun 350: 588–592. 17022935 10.1016/j.bbrc.2006.09.091 PMC1850102

[11] Roach PJ (2011) Are there errors in glycogen biosynthesis and is laforin a repair enzyme? FEBS Letters 585: 3216–3218. 21930129 10.1016/j.febslet.2011.09.009 PMC4939770

[12] Sakai M, Austin J, Witmer F, Trueb L (1970) Studies in myoclonus epilepsy (Lafora body form). II. Polyglucosans in the systemic deposits of myoclonus epilepsy and in corpora amylacea. Neurology 20: 160–176. 4188951 10.1212/wnl.20.2.160

[13] Schnabel R, Seitelberger F (1968) Histophysical and histochemical investigations of myoclonus bodies. Pathol Eur 3: 218–226. 4176997

[14] Tagliabracci VS, Girard JM, Segvich D, Meyer C, Turnbull J, et al (2008) Abnormal metabolism of glycogen phosphate as a cause for Lafora disease. J Biol Chem 283: 33816–33825. 18852261 10.1074/jbc.M807428200 PMC2590708

[15] Tagliabracci VS, Heiss C, Karthik C, Contreras CJ, Glushka J, et al (2011) Phosphate incorporation during glycogen synthesis and Lafora disease. Cell Metab 13: 274–282. 21356517 10.1016/j.cmet.2011.01.017 PMC3124772

[16] Nitschke F, Wang P, Schmieder P, Girard JM, Awrey DE, et al (2013) Hyperphosphorylation of glucosyl c6 carbons and altered structure of glycogen in the neurodegenerative epilepsy lafora disease. Cell Metab 17: 756–767. 23663739 10.1016/j.cmet.2013.04.006

[17] Gentry MS, Romá-Mateo C, Sanz P (2012) Laforin, a protein with many faces: glucan phosphatase, adapter protein, et alii. Febs J. 10.1111/j.1742-4658.2012.08549.x PMC3371293 22364389

[18] Romá-Mateo C, Sanz P, Gentry MS (2012) Deciphering the role of malin in the lafora progressive myoclonus epilepsy. IUBMB Life 64: 801–808. 22815132 10.1002/iub.1072 PMC3458166

[19] Chan EM, Bulman DE, Paterson AD, Turnbull J, Andermann E, et al (2003) Genetic mapping of a new Lafora progressive myoclonus epilepsy locus (EPM2B) on 6p22. J Med Genet 40: 671–675. 12960212 10.1136/jmg.40.9.671 PMC1735578

[20] Chan EM, Young EJ, Ianzano L, Munteanu I, Zhao X, et al (2003) Mutations in NHLRC1 cause progressive myoclonus epilepsy. Nat Genet 35: 125–127. 12958597 10.1038/ng1238

[21] Gentry MS, Worby CA, Dixon JE (2005) Insights into Lafora disease: malin is an E3 ubiquitin ligase that ubiquitinates and promotes the degradation of laforin. Proc Natl Acad Sci U S A 102: 8501–8506. 15930137 10.1073/pnas.0503285102 PMC1150849

[22] Vilchez D, Ros S, Cifuentes D, Pujadas L, Valles J, et al (2007) Mechanism suppressing glycogen synthesis in neurons and its demise in progressive myoclonus epilepsy. Nat Neurosci 10: 1407–1413. 17952067 10.1038/nn1998

[23] Cheng A, Zhang M, Gentry MS, Worby CA, Dixon JE, et al (2007) A role for AGL ubiquitination in the glycogen storage disorders of Lafora and Cori’s disease. Genes Dev 21: 2399–2409. 17908927 10.1101/gad.1553207 PMC1993871

[24] Rubio-Villena C, Garcia-Gimeno MA, Sanz P (2013) Glycogenic activity of R6; a protein phosphatase 1 regulatory subunit; is modulated by the laforin-malin complex. Int J Biochem Cell Biol. 10.1016/j.biocel.2013.04.019 23624058

[25] Solaz-Fuster MC, Gimeno-Alcaniz JV, Ros S, Fernandez-Sanchez ME, Garcia-Fojeda B, et al (2008) Regulation of glycogen synthesis by the laforin-malin complex is modulated by the AMP-activated protein kinase pathway. Hum Mol Genet 17: 667–678. 18029386 10.1093/hmg/ddm339

[26] Sengupta S, Badhwar I, Upadhyay M, Singh S, Ganesh S (2011) Malin and laforin are essential components of a protein complex that protects cells from thermal stress. J Cell Sci 124: 2277–2286. 21652633 10.1242/jcs.082800

[27] Rao SN, Sharma J, Maity R, Jana NR (2010) Co-chaperone CHIP stabilizes aggregate-prone malin, a ubiquitin ligase mutated in Lafora disease. J Biol Chem 285: 1404–1413. 19892702 10.1074/jbc.M109.006312 PMC2801266

[28] Mittal S, Dubey D, Yamakawa K, Ganesh S (2007) Lafora disease proteins malin and laforin are recruited to aggresomes in response to proteasomal impairment. Hum Mol Genet 16: 753–762. 17337485 10.1093/hmg/ddm006

[29] Aguado C, Sarkar S, Korolchuk VI, Criado O, Vernia S, et al (2010) Laforin, the most common protein mutated in Lafora disease, regulates autophagy. Hum Mol Genet 19: 2867–2876. 20453062 10.1093/hmg/ddq190 PMC2893813

[30] Criado O, Aguado C, Gayarre J, Duran-Trio L, Garcia-Cabrero AM, et al (2012) Lafora bodies and neurological defects in malin-deficient mice correlate with impaired autophagy. Hum Mol Genet 21: 1521–1533. 22186026 10.1093/hmg/ddr590

[31] Puri R, Ganesh S (2010) Laforin in autophagy: a possible link between carbohydrate and protein in Lafora disease? Autophagy 6: 1229–1231. 20818153 10.4161/auto.6.8.13307

[32] Vernia S, Rubio T, Heredia M, Rodriguez de Cordoba S, Sanz P (2009) Increased endoplasmic reticulum stress and decreased proteasomal function in lafora disease models lacking the phosphatase laforin. PLoS One 4: e5907. 19529779 10.1371/journal.pone.0005907 PMC2692001

[33] Alonso A, Rojas A, Godzik A, Mustelin T (2004) The dual-specific protein tyrosine phosphatase family. In: Ariño J, & Alexander, D.R., editor. Topics in Current Genetics: Protein Phosphatases. Heidelberg: Springer Berlin. 333–358.

[34] Pulido R, Hooft van Huijsduijnen R (2008) Protein tyrosine phosphatases: dual-specificity phosphatases in health and disease. Febs J 275: 848–866. 18298792 10.1111/j.1742-4658.2008.06250.x

[35] Wang J, Stuckey JA, Wishart MJ, Dixon JE (2002) A unique carbohydrate binding domain targets the lafora disease phosphatase to glycogen. J Biol Chem 277: 2377–2380. 11739371 10.1074/jbc.C100686200

[36] Ganesh S, Agarwala KL, Ueda K, Akagi T, Shoda K, et al (2000) Laforin, defective in the progressive myoclonus epilepsy of Lafora type, is a dual-specificity phosphatase associated with polyribosomes. Hum Mol Genet 9: 2251–2261. 11001928 10.1093/oxfordjournals.hmg.a018916

[37] Liu Y, Wang Y, Wu C, Zheng P (2006) Dimerization of Laforin is required for its optimal phosphatase activity, regulation of GSK3beta phosphorylation, and Wnt signaling. J Biol Chem 281: 34768–34774. 16971387 10.1074/jbc.M607778200

[38] Dukhande VV, Rogers DM, Romá-Mateo C, Donderis J, Marina A, et al (2011) Laforin, a dual specificity phosphatase involved in Lafora disease, is present mainly as monomeric form with full phosphatase activity. PLoS One 6: e24040. 21887368 10.1371/journal.pone.0024040 PMC3162602

[39] Romá-Mateo C, Solaz-Fuster Mdel C, Gimeno-Alcaniz JV, Dukhande VV, Donderis J, et al (2011) Laforin, a dual-specificity phosphatase involved in Lafora disease, is phosphorylated at Ser25 by AMP-activated protein kinase. Biochem J 439: 265–275. 21728993 10.1042/BJ20110150 PMC3299407

[40] Larkin MA, Blackshields G, Brown NP, Chenna R, McGettigan PA, et al (2007) Clustal W and Clustal X version 2.0. Bioinformatics 23: 2947–2948. 17846036 10.1093/bioinformatics/btm404

[41] Hall TA (1999) BioEdit: a user-friendly biological sequence alignment editor and analysis program for Windows 95/98/NT. Nucl Acids Symp Ser 95–98.

[42] Sorimachi K, Le Gal-Coeffet MF, Williamson G, Archer DB, Williamson MP (1997) Solution structure of the granular starch binding domain of Aspergillus niger glucoamylase bound to beta-cyclodextrin. Structure 5: 647–661. 9195884 10.1016/s0969-2126(97)00220-7

[43] Vander Kooi CW, Taylor AO, Pace RM, Meekins DA, Guo HF, et al (2010) Structural basis for the glucan phosphatase activity of Starch Excess4. Proc Natl Acad Sci U S A 107: 15379–15384. 20679247 10.1073/pnas.1009386107 PMC2932622

[44] Pei J, Kim BH, Grishin NV (2008) PROMALS3D: a tool for multiple protein sequence and structure alignments. Nucleic Acids Res 36: 2295–2300. 18287115 10.1093/nar/gkn072 PMC2367709

[45] Arnold K, Bordoli L, Kopp J, Schwede T (2006) The SWISS-MODEL workspace: a web-based environment for protein structure homology modelling. Bioinformatics 22: 195–201. 16301204 10.1093/bioinformatics/bti770

[46] Luthy R, Bowie JU, Eisenberg D (1992) Assessment of protein models with three-dimensional profiles. Nature 356: 83–85. 1538787 10.1038/356083a0

[47] Christen M, Hunenberger PH, Bakowies D, Baron R, Burgi R, et al (2005) The GROMOS software for biomolecular simulation: GROMOS05. J Comput Chem 26: 1719–1751. 16211540 10.1002/jcc.20303

[48] Vernia S, Solaz-Fuster MC, Gimeno-Alcaniz JV, Rubio T, Garcia-Haro L, et al (2009) AMP-activated protein kinase phosphorylates R5/PTG, the glycogen targeting subunit of the R5/PTG-protein phosphatase 1 holoenzyme, and accelerates its down-regulation by the laforin-malin complex. J Biol Chem 284: 8247–8255. 19171932 10.1074/jbc.M808492200 PMC2659182

[49] Paumi CM, Menendez J, Arnoldo A, Engels K, Iyer KR, et al (2007) Mapping protein-protein interactions for the yeast ABC transporter Ycf1p by integrated split-ubiquitin membrane yeast two-hybrid analysis. Mol Cell 26: 15–25. 17434123 10.1016/j.molcel.2007.03.011

[50] Ludin K, Jiang R, Carlson M (1998) Glucose-regulated interaction of a regulatory subunit of protein phosphatase 1 with the Snf1 protein kinase in Saccharomyces cerevisiae. Proc Natl Acad Sci U S A 95: 6245–6250. 9600950 10.1073/pnas.95.11.6245 PMC27646

[51] Gentry MS, Pace RM (2009) Conservation of the glucan phosphatase laforin is linked to rates of molecular evolution and the glucan metabolism of the organism. BMC Evol Biol 9: 138. 19545434 10.1186/1471-2148-9-138 PMC2714694

[52] Liu Y, Wang Y, Wu C, Zheng P (2009) Deletions and missense mutations of EPM2A exacerbate unfolded protein response and apoptosis of neuronal cells induced by endoplasm reticulum stress. Hum Mol Genet 18: 2622–2631. 19403557 10.1093/hmg/ddp196 PMC2701334

[53] Christiansen C, Abou Hachem M, Janecek S, Vikso-Nielsen A, Blennow A, et al (2009) The carbohydrate-binding module family 20–diversity, structure, and function. Febs J 276: 5006–5029. 19682075 10.1111/j.1742-4658.2009.07221.x

[54] Dukhande VV, Rogers DM, Romá-Mateo C, Donderis J, Marina A, et al (2011) Laforin, a dual specificity phosphatase involved in Lafora disease, is present mainly as monomeric form with full phosphatase activity. PLoS One 6: e24040. 21887368 10.1371/journal.pone.0024040 PMC3162602

[55] Sherwood AR, Paasch BC, Worby CA, Gentry MS (2013) A malachite green-based assay to assess glucan phosphatase activity. Anal Biochem 435: 54–56. 23201267 10.1016/j.ab.2012.10.044 PMC3577954

[56] Kaiser P, Tagwerker C (2005) Is this protein ubiquitinated? Methods Enzymol 399: 243–248. 16338360 10.1016/S0076-6879(05)99016-2

[57] Koksal AC, Cingolani G (2011) Dimerization of Vaccinia virus VH1 is essential for dephosphorylation of STAT1 at tyrosine 701. J Biol Chem 286: 14373–14382. 21362620 10.1074/jbc.M111.226357 PMC3077637

