# Supplementary material for: Dimerization of the Glucan Phosphatase Laforin Requires the Participation of Cysteine 329
Source: PLoS One. 2013 Jul 26;8(7):e69523. doi: 10.1371/journal.pone.0069523 (PMC3724922; doi:10.1371/journal.pone.0069523)
Supplement: Table S1 — List of entries used for sequence analysis. Left column shows the UniprotKB ID label for the laforin orthologs used in the sequence alignment. Right column shows the species name corresponding to each protein ID. (DOCX) [file pone.0069523.s001.docx]

**Supplementary Table S1. List of entries used for sequence analysis**

| **UniprotKB ID** | **SPECIES** |
| --- | --- |
| sp\|O95278\|EPM2A_HUMAN | *Homo sapiens* |
| tr\|A5PK37\|A5PK37_BOVIN EPM2A | *Bos taurus* |
| sp\|Q1M199\|EPM2A_CANFA | *Canis familiaris* |
| sp\|Q91XQ2\|EPM2A_RAT | *Rattus norvegicus* |
| sp\|Q9WUA5\|EPM2A_MOUSE | *Mus musculus* |
| tr\|Q5ZL46\|Q5ZL46_CHICK | *Gallus gallus* |
| tr\|Q6GPD8\|Q6GPD8_XENLA | *Xenopus laevis* |
| tr\|Q4S6Z3\|Q4S6Z3_TETNG | *Tetraodon nigroviridis* |
| tr\|A7SVW9\|A7SVW9_NEMVE | *Nematostella vectensis* |
| tr\|B6KIB6\|B6KIB6_TOXGO | *Toxoplasma gondii* |
