# Supplementary material for: Dimerization of the Glucan Phosphatase Laforin Requires the Participation of Cysteine 329
Source: PLoS One. 2013 Jul 26;8(7):e69523. doi: 10.1371/journal.pone.0069523 (PMC3724922; doi:10.1371/journal.pone.0069523)
Supplement: Table S2 — Primers used for mutagenesis analysis. Mutagenesis was performed by PCR reactions combining laforin terminal primers (upper panel) with internal primers harboring each mutation (down panel). Sequences are written in 5′–3′ sense. (DOCX) [file pone.0069523.s002.docx]

**Supplementary Table S2. Primers used for mutagenesis analysis**

| **LAFORIN TERMINAL PRIMER (FORWARD)** | **LAFORIN TERMINAL PRIMER**  **(REVERSE)** |  |
| --- | --- | --- |
| GGGAATTCCATCTGCGCTTCCGCTTTGGGGTG | ATAAGAATGCGGCCGCACTACAGGCTACACACAGAAGAACG |  |

| **FORWARD PRIMER** | **REVERSE PRIMER** | **MUTATION** |
| --- | --- | --- |
| AATGGACCTCATCATGACCGTTCCTCTACTTACAATGAAAACAACTTG | CAAGTTGTTTTCATTGTAAGTAGAGGAACGGTCATGATGAGGTCCATT | C109S -C110S |
| AACTTGGTGGATGGTGTGTATTCTCTCCCAATAGGACACTGGATT | AATCCAGTGTCCTATTGGGAGAGAATACACACCATCCACCAAGTT | C123S |
| CCAAATATCTGGCTGGGTAGCTCCCCTCGTCAGGTGGAACATGTA | TACATGTTCCACCTGACGAGGGGAGCTACCCAGCCAGATATTTGG | C169S |
| ATTGTACAGAATTCCTCAGGCTCTAACCGCTACCCAGAGCCCATG | CATGGGCTCTGGGTAGCGGTTAGAGCCTGAGGAATTCTGTACAAT | C205S |
| CAGATGCTGCCCCAGGCGGTGTCCCTGCTGCATGCGCTGCTGGAG | CTCCAGCAGCGCATGCAGCAGGGACACCGCCTGGGGCAGCATCTG | C250S |
| GGACACATCGTGTACGTGCACTCCAACGCTGGGGTGGGCCGCTCC | GGAGCGGCCCACCCCAGCGTTGGAGTGCACGTACACGATGTGTCC | C266S |
| GGCCGCTCCACCGCGGCTGTCTCCGGCTGGCTCCAGTATGTGATG | CATCACATACTGGAGCCAGCCGGAGACAGCCGCGGTGGAGCGGCC | C278S |
| GGGAATTCCATCTGCGCTTCCGCTTTGGGGTG | ATAAGAATGCGGCCGCACTACAGGCTAGACACAGAAGAACG | C329S |
